# Applications of Artificial Intelligence in Ophthalmology: General Overview

**DOI:** 10.1155/2018/5278196

**Published:** 2018-11-19

**Authors:** Wei Lu, Yan Tong, Yue Yu, Yiqiao Xing, Changzheng Chen, Yin Shen

**Affiliations:** ^1^Eye Center, Renmin Hospital of Wuhan University, Eye Institute of Wuhan University, Wuhan, Hubei, China; ^2^Hisee Medical Artificial Intelligent Lab, Wuhan University, Wuhan, Hubei, China

## Abstract

With the emergence of unmanned plane, autonomous vehicles, face recognition, and language processing, the artificial intelligence (AI) has remarkably revolutionized our lifestyle. Recent studies indicate that AI has astounding potential to perform much better than human beings in some tasks, especially in the image recognition field. As the amount of image data in imaging center of ophthalmology is increasing dramatically, analyzing and processing these data is in urgent need. AI has been tried to apply to decipher medical data and has made extraordinary progress in intelligent diagnosis. In this paper, we presented the basic workflow for building an AI model and systematically reviewed applications of AI in the diagnosis of eye diseases. Future work should focus on setting up systematic AI platforms to diagnose general eye diseases based on multimodal data in the real world.

## 1. Introduction

As population aging has become a major demographic trend around the world, patients suffering from eye diseases are expected to increase steeply. Early detection and appropriate treatment of eye diseases are of great significance to prevent vision loss and promote living quality. Conventional diagnose methods are tremendously depend on physicians' professional experience and knowledge, which lead to high misdiagnosis rate and huge waste of medical data. Deep integration of ophthalmology and artificial intelligence (AI) has the potential to revolutionize current disease diagnose pattern and generate a significant clinical impact.

Proposed in 1956 by Dartmouth scholar John McCarthy, AI is a general term that “refers to hardware or software that exhibits behavior which appears intelligent” [1]. Though occurred sixty years ago, it is until recently that the effectiveness of AI has been highlighted because of the development of new algorithms, specialized hardware, cloud-based services, and big data. Machine learning (ML), occurred in 1980s, is a subset of AI, and is defined as a set of methods that automatically detect patterns in data and then incorporate this information to predict future data under uncertain conditions. Deep learning (DL), occurred in 2000s, is a burgeoning technology of ML and has revolutionized the world of AI. These technologies power many aspects of modern society, such as objects' recognition in images, real-time languages' translation, device manipulation via speech (such as Apple's Siri, Amazon Alexa, and Microsoft Cortana), and so on.

The field of healthcare has been the forefront of the AI application in recent years. Multiple studies have shown that DL algorithms performed at a high level when applied to breast histopathology analysis [2], skin cancer classification [3], cardiovascular diseases' risk prediction [4], and lung cancer detection [5]. These impressive research studies inspire numerous studies to apply AI in ophthalmology. Advanced AI algorithms together with multiple accessible data sets, such as EyePACS [6], Messidor [6], and Kaggle's data set [7], can make breakthroughs on various ophthalmological issues.

The rapid rise in AI technology requires physicians and computer scientists to have a good mutual understanding of not only the technology but also the medical practice to enhance medical care in the near future. Miguel Caixinha and Sandrina Nunes introduced conventional machine learning (CML) techniques and reviewed applications of CML for diagnosis and monitoring of multimodal ocular disease, without the mention about DL [8]. Litjens et al. [9] detailly introduced various DL methods for different tasks and provided an overview of studies per application area, whereas the “retina” section majorly focused on the fundus images only. Lee et al. [10] introduced the AI development in ophthalmology generally. Rahimy [11] focused on DL applications in the ophthalmology field, without the mention about CML. Louis J. Catania and Ernst Nicolitz systemically reviewed AI and robotic applications in multiple categories of vision and eye care but mentioned little about AI diagnosis of retinal diseases [12].

In this review, we systematically reviewed the application of AI (both CML and DL) in diagnosing ocular diseases, including the four leading cause of adult blindness diabetic retinopathy (DR), glaucoma, age-related macular degeneration (AMD), and cataract. We also introduced the existing AI methods, the ophthalmic imaging modalities, detailed steps for building AI models, and evaluation metrics in AI diagnosis. We hope we can provide both ophthalmologists and computer scientists a meaningful and comprehensive summary on AI applications in ophthalmology and facilitate promising AI projects in the ophthalmology field.

## 2. AI Algorithms

**Figure 1:**
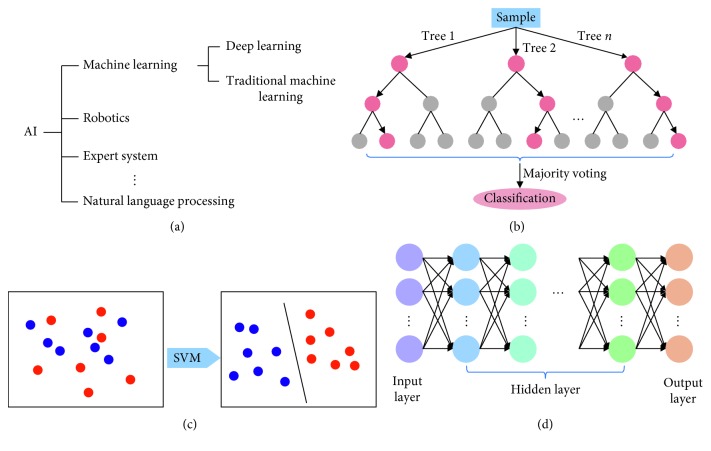
Introduction of AI algorithms. (a) The relationship among AI, ML, and DL. (b) The workflow of a RF. (c) The principle of an SVM. (d) The schematic diagram of a typical deep neural network.

As we mentioned above, ML is one subset of AI and includes DL and CML (Figure 1(a)). The defining characteristic of ML algorithms is the quality of predictions improved with experience [13]. The more data we provide (usually up to a platform), the better the prediction model we can achieve.

Supervised learning and unsupervised learning are two forms of ML. Supervised learning is to train a model from already labeled training data, tunes the weightings of the inputs to improve the accuracy of its predictions until they are optimized, and then map test data sets as corresponding outputs. It may expedite classification process and would be useful for discriminating clinical outcomes. Unsupervised learning is to train a model with unlabeled data (without a human-labeled process), infers a function to describe hidden structures that usually invisible to humans, and could bring new discoveries, such as new encephalic region relevant to Alzheimer's disease [14] and new impact factors of cardiovascular diseases beyond human's recognition [4]. So far, methods adopted in most research studies are in supervised form because the accuracy and efficacy are better under supervised condition [15].

CML can get satisfactory outcome with small data sets, but a cumbersome step to select specific visual features manually prior to classification is indispensable [16]. This selection can result in a set of suboptimal features and overfitting (the trained model is not generalized to other data except for the training set), which limits CML algorithms' application.

Existing CML algorithms used in AI diagnosis include decision trees [17], random forests (RF) [18], support vector machines (SVM) [19], Bayesian classifiers [20], k-nearest neighbors [21], k-means [22], linear discriminant analysis [23], and neural networks (NN) [24] (Table 1). Among them, RF and SVM are the most commonly used CML technologies in the ophthalmology field [25] (Figures 1(b) and 1(c)).

DL, a burgeoning technology of ML, has the ability to discover intricate structures in data sets without the need to specify rules explicitly. A DL network is an NN with multiple layers between the input and output layers (Figure 1(d)). It has dramatically improved the state-of-the-art in image recognition [15]. When applied to image classification, a key difference between DL and CML algorithms is how they select and process image features. Features of input data are automatically learned in an unsupervised way by DL algorithms, avoiding manual segmenting and depicting lesions' areas [15, 26]. However, large data set is needed to train a DL algorithm. Transfer learning is to retrain an algorithm, which has already been pretrained on millions of general images before, on a specific data set. This method allows the training of a highly accurate model with a relatively small training data set [27].

DL algorithms are known as “black boxes.” The networks generate comprehensive and discriminative features that are much too high dimensional to be accessible for human interpretation. Little is known about how they analyze pattern and make a decision at the image level [7]. Heatmaps can show which pixels play a role in the image-level predictions. In the medical field, the visualization highlighted highly possible abnormal regions in the input image for future review and analysis, potentially aiding real-time clinical validation of automated diagnoses at the point of care. Existing methods of DL include long-term and short-term memory [15], deep Boltzmann machines [28], deep kernel machines [29], deep recurrent neural networks [30], and convolutional neural networks (CNN) [15]. Among them, the most used DL method in the medical image recognition field is CNN. The CNN consists of multiple convolutional layers that extract features and transform input images into hierarchical feature maps: from simple features, such as edges and lines, to complicated features, such as shapes and colors. It also includes layers that can merge semantically similar features into one to reduce the dimensionality of the extracted features, and layers that can combine these features and output a final probability value for the class. Existing CNN architectures used in the medical image recognition field include AlexNet [31], VGG [32], ResNet [33], and GoogleNet [34–37](Table 2).

## 3. Building AI Models

Various imaging modalities have been used in AI diagnosis, such as radiology images (X-ray, CT, and MRI) [38], electrophysiological signal records (electrocardiograph [39] and electroencephalogram [40]), visible wavelength images (dermoscopy images and biopsy images [3]), ultrasound images [41], angiography images [42], and so on. We introduce the ophthalmic imaging modalities in AI diagnosis in Table 3.

The steps for building an AI model include preprocessing image data, train, validate and test the model, and evaluate the trained model's performance.

### 3.1. Data Preprocessing

In order to increase AI prediction efficiency, raw data need to be preprocessed. The preprocessed work includes the following [8, 43]: (1) noise reduction: noise reduction needs to be performed in almost all relevant research. Denoising can promote the quality of data set and optimize learning process. (2) Data integration and normalization: data collected from different sources should be integrated and adjusted to a common scale. (3) Feature selection and extraction: the most relevant features are usually selected and extracted to improve the learning process performance.

### 3.2. Training, Validation, and Test

**Figure 2:**
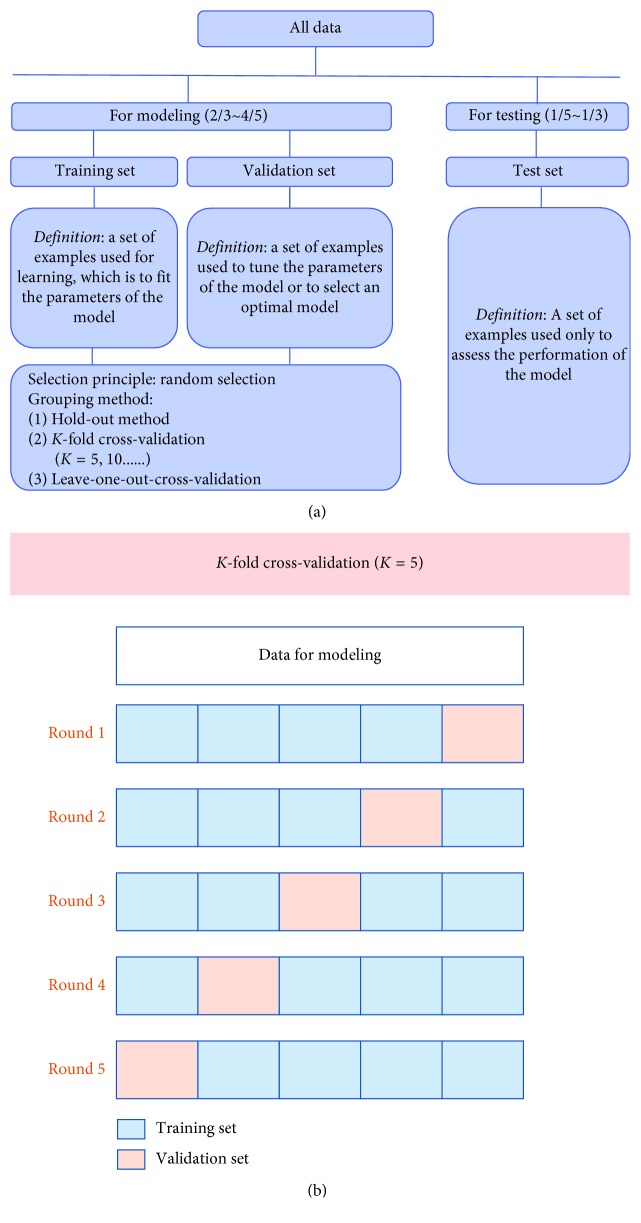
Data partitioning method during data processing. (a) A brief introduction of data partition. (b) An illustration of a specific process of 5-fold cross-validation.

To achieve a good performance, the data set is randomly partitioned into two independent subsets, one is for modeling and the other is for testing. The data in the former sets will be partitioned again into training set and validation set in most cases. The training set is used to fit the parameters of a model. The validation set is used to estimate how well the model had been trained and tune the parameters or to compare the performances of the prediction algorithms achieved based on the training set. The test set is used to evaluate the final performance of the trained model (Figure 2(a)).

Cross-validation methods have been widely used to estimate and optimize algorithms [44]. The most adopted cross-validation is “K-fold cross-validation.” It is an effective method to avoid overfitting and underfitting. All data are equally divided into K subsets, 1 for validation and K − 1 for training. This process will repeat K times, and average metrics are used to evaluate the trained model (Figure 2(b)). Fivefold cross-validation and 10-fold cross-validation are most commonly used [44].

### 3.3. Evaluation

Receiver operating characteristic curve (ROC) is a useful tool to depict algorithms' performance. It is created by plotting the detection probability for each algorithm across a continuum of threshold. For each threshold, the sensitivity and the false positive rate (1 − specificity) are plotted against each other. The area under receiver operating characteristic curves (AUC) is the most used evaluation metrics for quantitative assessment of a model in AI diagnosis. The AUCs of effective models range from 0.5 to 1; the higher the value of AUC, the better the performance of the model [45].  Table 4 provides introduction of other metrics to evaluate the performance of a model.

## 4. AI Application in Ophthalmology

**Figure 3:**
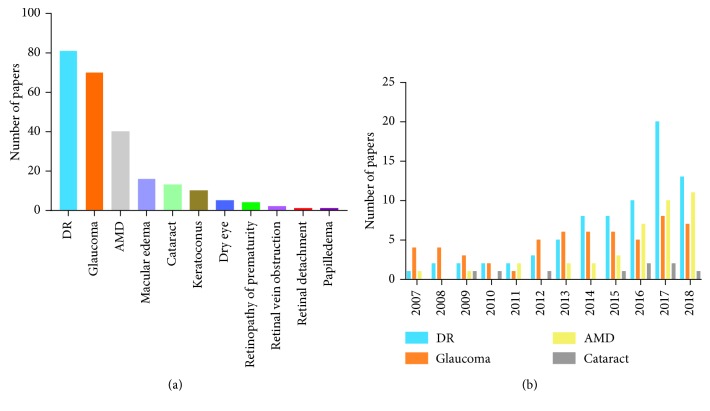
Publication of AI application in diagnosing ophthalmological diseases. (a) Publication statistics per ophthalmological diseases. (b) Publication statistics per year (Jan 1, 2007 to Sep 20, 2018).

Two hundred forty-three articles of AI application in diagnosing ophthalmological diseases have been published (search by PubMed, Sep 20, 2018). Among them, the most intensively studied are DR, glaucoma, AMD, and cataract (Figure 3(a)).  Figure 3(b) shows the breakdown of the papers of these four diseases in year of publication.

### 4.1. Diabetic Retinopathy

Diabetes affects more than 415 million people worldwide, meaning 1 in every 11 adults is affected [46]. DR, a chronic diabetic complication, is a vasculopathy that affects one-third of diabetic patients and can lead to irreversible blindness [47]. Automated techniques for DR diagnosis have been explored to improve the management of patients with DR and alleviate social burden. AI was used to predict DR risk and DR progression among diabetic patients to combat with this worldwide disease [48, 49].

The specific abnormalities such as macular edema [50–53], exudates [53], cotton-wool [54], microaneurysms [55, 56], and neovascularization on optic disk [57] can be detected by CML. Based on these hallmarks, the early diagnose of DR in an automated fashion has been explored [58]. Additionally, a system focused on timely and effectively proliferative DR (PDR) detection has been developed to ensure immediate attention and intervention [59, 60].

Gulshan et al. were the first to report the application of DL for DR identification [6]. They used large fundus image data sets to train a deep CNN (DCNN) in a supervised manner. They showed that the method based on DL techniques had very high sensitivity and specificity, and the AUC came up to 0.99 for detecting referable DR [61]. In the past two years, a number of DL models with impressive performance have been developed for the automated detection of DR [46, 62, 63]. Additionally, some studies applied DL to automatically stage DR through fundus images [62–65], making up the deficiency of Gulshan's study that they only detected referable DR but did not provide comparable data on sight-threatening DR or other DR stages.

The majority of aforementioned studies focused mainly on the analysis of fundus photographs. There were some other imaging modalities used to build models for DR. ElTanboly et al. developed a DL-based computer-aided system to detect DR through 52 optical coherence tomography (OCT) images, achieving an AUC of 0.98 [66]. Despite the good outcomes in the cross-validation process, the system needs to be further validated in larger patient cohorts. A computer-aided diagnostic (CAD) system based on CML algorithms using optical coherence tomography angiography (OCTA) images to automatically diagnose nonproliferative DR (NPDR) also achieved high accuracy and AUC [67].

The visualization of which pixels play an important role in the image-level predictions has been applied into DR diagnostic models [7, 46]. It represents intuitively the learning procedure of the DL network and highlights important abnormal regions, assisting physicians' better understanding of the DR predictions. The visualization method can enhance the applicability of intelligent diagnostic models in real clinical practice.

### 4.2. Glaucoma

Glaucoma is the third largest sight-threatening eye disease around the world and has critical impact on global blindness [68]. Glaucoma patients suffered from high intraocular pressure, damage of the optic nerve head (ONH), retina nerve fiber layer (RNFL) defect, and gradual vision loss. Automatically detecting features related to glaucoma has great significance on its timely diagnosis.

The optic cup-to-disc ratio (CDR) can be used to detect glaucoma patients [69]. Based on automatically localization of ONH and extraction of optic disc and optic cup from fundus images [70], CDR can be calculated to assist glaucoma diagnose at an early stage by AI models [71–74]. Spectrum domain OCT (SD-OCT) is another imaging modality to evaluate CDR. After approximately locating the coarse disc margin by a spatial correlation smoothness constraint, a SVM model is trained to find the most likely patch on OCT images to determine a reference plane that can calculate the CDR. The proposed algorithm can achieve high segmentation accuracy and a low CDR evaluation error [75].

RNFL defect can serve as the earliest sign of glaucoma [76]. Several researchers have explored diagnostic accuracy of different methods using RNFL thickness parameters to diagnose glaucoma [77–79]. However, high myopia patients can also suffer from RNFL thickness reduction [80–83]. Recently, reports on how to distinguish the normal retina from glaucoma in high myopia via OCT parameters and optic disc morphology have been published. This indicates us to take account into the existence of other eye diseases in future's research about glaucoma's intelligent diagnosis to improve the accuracy of algorithms.

Visual field (VF) defect is a main alteration of visual function during glaucoma progress. Recent studies showed that changes in the central visual field may already occurred in the early stage of the disease, which is consistent with the results of imaging studies [84]. Thus, the early detection of glaucomatous VF changes is significant to glaucoma's successful detection and management [85]. Applying ML methods can improve the detection of preperimetric glaucoma VFs from healthy VFs significantly [86]. Although a standard automated VF test plays a key role in diagnosing glaucoma, it consumes too much time and resources. What is more, such a manual process performed by patients is subjective and has shown strong variability in epidemiologic studies [87]. The combination of all features mentioned above is required for the accurate intelligent diagnosis, for any of the single symptom is not the guarantee sign of glaucoma [83, 88]. This kind of research shows great performance in classifying glaucoma and healthy eyes. Clinicians may reference these prediction results and make better decisions.

Studies using DL methods to diagnose glaucoma are few. So far, fundus images [73, 89, 90], VFs [91], and wide-field OCT scans [92] have all been used to construct DL-based glaucomatous diagnostic models. Preperimetric open-angle glaucoma (OAG) eyes can be detected through DL with better performance than those got from CML techniques [91]. Holistic and local features of optic disc on fundus images have been used together to mitigate the influence of misalignment when located optic disc for glaucoma diagnosis [89]. The AUC was 0.8384, which is quite close to the manual detection results. Li et al. demonstrated that DL can be applied to identify referable glaucomatous optic neuropathy with high sensitivity and specificity [90].

### 4.3. Age-Related Macular Degeneration

AMD is the leading cause of irreversible blindness among old people in the developed world [93]. The goal of using ML algorithms is to automatically identify AMD-related lesions to improve AMD diagnosis and treatment. Detection of drusen [93, 94], fluid [94, 95], reticular pseudodrusen [96], and geographic atrophy [97] from fundus images and SD-OCT using ML [96] has been studied. The accuracy is usually over 80% [93, 96–98], and the agreement between the models and retina specialists can reach 90%.

Drusen regression, an anatomic endpoint of intermediate AMD and the onset of advanced AMD, can be predicted through the specifically designed, fully automated, ML-based classifier. Bogunovic et al. develop a data-driven interpretable predictive model to predict the progression risk in intermediate AMD [94]. Automated image analysis steps were applied to identify and characterize individual drusen at baseline, and their development was monitored at every follow-up visit. Using such characterization and analysis, they developed an ML method based on survival analysis to estimate a risk score and predict the incoming regression of individual drusen. Above all, these automated detections of the retinal lesions combined with interpretation of disease activity are feasible and have the potential to become a powerful tool in clinical practice [95].

Using ML to predict anti-vascular endothelial growth factor (anti-VEGF) injection requirements in eye diseases such as neovascular AMD and PDR can alleviate patients' economic burden and facilitate resource management. Bogunovic et al. fed corresponding OCT images of patients with low or high anti-VEGF injection requirements into RF to obtain a predictive model. A solid AUC of 70% to 80% was achieved for treatment requirement prediction [99]. Prahs et al. trained a DCNN neural network by OCT images to facilitate decision-making regarding anti-VEGF injection [100], and the outcomes were better than that of using CML [99]. These studies are an important step toward image-guided prediction of treatment intervals in the management of neovascular AMD or PDR.

Multiple CML techniques have been applied for automated diagnosis and grading of AMD [101, 102]. But the most impressive work was based on DL techniques over the past 2 years [103–105]. Treder et al. establish a model to automatically detect exudative AMD from SD-OCT [105]. In research studies based on fundus images, images with AMD were assigned into 4 classes of classification (no evidence of AMD, early-stage AMD, intermediate-stage AMD, and advanced AMD) [104], or 2-class classification (no or early-stage AMD and intermediate or advanced stage AMD) [103]. The diagnostic accuracy is better in the 2-class classification in current studies. The DCNN appears to perform a screening function in these experiments, and the performance is comparable with physicians. DL algorithms have also been used to automatically detect abnormalities such as exudates [106], macular edema [51, 52], drusen, and choroidal neovascularization [27].

### 4.4. Cataract

Cataract is a disease with cloudy lens and has bothered millions of old people. Early detection and treatment can bring the light to cataract patients and improve their living quality. ML algorithms such as RF and SVM have been applied to diagnose and grading cataract from fundus images, ultrasounds images, and visible wavelength eye images [107–109]. The risk prediction model for posterior capsule opacification after phacoemulsification has also been built [110].

Researchers can now use DL models to diagnose senile cataract [111], but a more impressive work is about the pediatric cataract. It is one of the primary causes of childhood blindness [112]. Long et al. constructed a CNN-based computer-aided diagnosis (CAD) framework to classify and grade pediatric cataract. What is more, a cloud-based platform integrated the AI agent for multihospital collaboration has been established. They even developed a software to realize clinical application for ophthalmologists and patients and have applied it in Zhong Shan Ophthalmic Center [113, 114]. These proposed methods are serviceable for improving clinical workflow of cataract's diagnosis in the background of large-population screening and mainly shed a light on other ocular images.

In addition to DR, glaucoma, AMD, and cataract, AI has also been used to diagnose other eye diseases. AI algorithms can be used to detect keratoconus or identify eyes with preclinical signs of keratoconus using data from a Scheimpflug camera [115, 116], to evaluate corneal power after myopic corneal refractive surgery [117], to make surgical plans for horizontal strabismus [118], and to detect pigment epithelial detachment in polypoidal choroidal vasculopathy [119].

Previous studies have summarized articles about the application of CML techniques in eye diseases [8]. In this review, we summarized studies on glaucoma, DR, AMD, and cataract using DL techniques in Table 5.

## 5. Future of AI Application in Clinic

In recent years, AI techniques have shown to be an effective diagnostic tool to identify various diseases in healthcare. Applications of AI can make great contributions to provide support to patients in remote areas by sharing expert knowledge and limited resources. While the accuracy of the models is incredible promising, we need to remain prudent and sober when considering how to deploy these models to the real world.

Most studies regarding intelligent diagnosis of eye diseases focused on binary classification problems, whereas in clinical setting, visiting patients suffer from multicategorical retinal diseases. For instance, a model trained to detect AMD will fail to consider a patient with glaucoma as diseased because the model only has the ability to discriminate AMD from non-AMD. Choi and his colleagues carried out a work applying DL to automatically detect multiple different retinal diseases with fundus photographs. When only normal and DR fundus images were involved in the proposed DL model, the classification accuracy was 87.4%. However, the accuracy fell to 30.5% when all 10 categories were included [120]. It indicated that the model's accuracy declined while the number of diseases increased. To further enhance the applicability of AI in clinic practice, we should make more efforts to build intelligent systems that can detect different retinal diseases with high accuracy.

Additionally, a single abnormality detected from one imaging technique cannot always guarantee the correct diagnosis of a specific retinal disease (e.g., DR or glaucoma) in clinical practice. Multimodal clinical images, such as optical coherence tomography angiography, visual field, and fundus images, should be integrated together to build a generalized AI system for more reliable AI diagnosis.

However, the need of huge amount of data remains the most fundamental problem. Although various data sets have been available, they only incorporate a small part of diseases human suffered from. Images with severe diseases or rare diseases are particularly insufficient. The population characteristics, the existence of various systematic diseases, and the diverse disease' phenotypes should be considered when select input data. Larger data sets from larger patient cohorts under different settings and conditions, such as diverse ethnics and environments, are also needed in some automated diagnosis systems with impressive outcomes for further validation.

The high dependency on the data quality should be considered. Different imaging devices, various imaging protocols, and intrinsic noise of data can affect the data's quality, which may have huge influences on models' performance [38]. In addition to data preprocessing, universal useful methods to analyze images with different qualities need to be developed urgently.

Although the DL-based methods show excellent results most of the time, their “black box” nature makes it difficult to interpret how algorithms make decisions. In this era of “evidence-based medicine,” it is difficult for clinicians and patients to trust a mysterious machine that cannot provide explanations of why the patient is diagnosed with a certain disease. What is more, the techniques that make the AI models more transparent can also detect potential bias in the training data and ensure that the algorithms perform well [121]. Heatmaps and the occlusion test are two of these kinds of techniques that can highlight highly possible abnormal regions for predictions and make models interpretable to some extent [7, 27]. More methods to interpret AI models should be developed and applied in AI diagnosis. Moreover, the standards to systematically assess these methods should also be considered and developed.

Above all, by building interpretable systematic AI platforms using sufficient high-quality and multimodal data and advanced techniques, we can enhance the applicability of AI in clinical circumstances. In some day, we might make it possible to adopt intelligent systems in certain process of clinical work. Though ethical, regulatory, and legal issues arise, AI will contribute remarkably to revolutionize current disease diagnostic pattern and generate a significant clinical impact in the near future.

## Figures and Tables

**Table 1:** Introduction of existing CML techniques in the AI medical field.

Classifiers	Principles
Decision trees	(i) Tree-like structure
	(ii) Solve classification and regression problems based on rules to binary split data
Random forests	(i) Ensemble a multitude of decision trees for classification
	(ii) The ultimate prediction is made by majority voting
Support vector machines	Build a hyperplane that separates the positive and negative examples as wide as possible to minimize the separation error
Bayesian classifiers	(i) Based on the probability approach
	(ii) Assign a new sample to the category with maximum posterior probability, depending on the given prior probability, cost function, and category conditional density
k-nearest neighbors	Search for k-nearest training instances and classify a new instance into the most frequent class of these *k* instances
k-means	Partition *n* samples into *k* clusters in which each sample belongs to the cluster with the nearest mean
Linear discriminant analysis	(i) Create predictive functions that maximize the discrimination between previously established categories
Neural networks	(i) Consists of a collection of connected units, which can process signals
	(ii) Connections between them can transmit a signal to another
	(iii) Units are organized in layers
	(iv) Signals travel from the input layer to the output layer

**Table 2:** Concise introduction of CNN algorithms used in AI diagnosis.

Models	Layers	Top-5 error^*∗*^ (%)	ILSVRC^*#*^
AlexNet (2012)	8 layers	15.3	2012
VGG (2014)	19 layers	7.3	2014
ResNet-152 (2015)	152 layers	3.57	2015
ResNet-101	101 layers	4.6	—
ResNet-50	50 layers	5.25	—
ResNet-34	34 layers	5.6	—
GoogleNet/inception v1 (2014) [34]	22 layers	6.7	2014
Inception v2 (2015) [35]	33 layers	4.8	—
Inception v3 (2015) [36]	47 layers	3.5	—
Inception v4 (2016) [37]	77 layers	3.08	—

^*∗*^The fraction of test images for which the correct label is not among the five labels considered most probable by the algorithm. The lower the top-5 error, the better the classifier perform. ^#^ImageNet large-scale visual recognition challenge.

**Table 3:** The ophthalmic imaging modalities in AI diagnosis.

Imaging modalities	Image features	Applications
Fundus image	Show a magnified and subtle view of the surface of the retina	Retinal diseases diagnose
Optical coherence tomography	Show micrometer-resolution, cross-sectional images of the retina	Retinal diseases diagnose
Ocular ultrasound B-scan	Show a rough cross-sectional view of the eye and the orbit	Evaluate the condition of lens, vitreous, retina, and tumor
Slit-lamp image	Provides a stereoscopic magnified view of the anterior segment in detail	Anterior segment diseases diagnose
Visual field	Show the size and shape of field-of-view	To find disorders of the visual signal processing system that includes the retina, optic nerve, and brain

**Table 4:** Introduction of metrics to evaluate the performance of a model.

Metrics	Definitions
Accuracy	Measure the proportion of samples that are correctly identified by a classifier among all samples
Sensitivity/recall rate	The number of actual positives divided by the number of all samples that have been identified as positive by a gold standard
Specificity	The number of actual negatives divided by the number of all samples that have been identified as negative by a gold standard
Precision/positive predictive value	The number of actual positives divided by the number of all positives identified by a classifier
Kappa value	To examine the agreement between a model with the ground truth on the assignment of categories
Dice coefficient/F1 score	Harmonic average of the precision and recall, where a F1 score reaches its best value at 1 (perfect precision and recall) and worst at 0

**Table 5:** Studies on eye diseases using DL techniques.

Groups	Aim	Data sets	Deep learning techniques	Performance	Conclusions
Gulshan et al. [6] (Google Inc.)	DR detection	Public: EyePACS, Messidor 128175 fundus images	DCNN	AUC 0.991 for EyePACS 0.990 for Messidor	The DCNN had high sensitivity and specificity for detecting referable DR (moderate and worse DR, referable diabetic macular edema, or both)

Gargeya and Leng [46] (Byers Eye Institute at Stanford)	DR detection	Public: EyePACS, Messidor, E-ophtha 77348 fundus images	DCNN	AUC 0.94 for Messidor data set 0.95 for E-ophtha data set	The DCNN can be used to screen fundus images to identify DR with high reliability

Quellec et al. [7] (Brest University)	DR detection heatmaps creation	Public: Kaggle, DiaretDB, E-ophtha 196590 fundus images	CNN	AUC = 0.954 in Kaggle's data set AUC = 0.949 in E-ophtha data set	The proposed method is a promising image mining tool to discover new biomarkers in images. The model trained to detect referable DR can detect some lesions and outperforms recent algorithms trained to detect those lesions specifically

Ardiyanto et al. [63] (Universitas Gadjah Mada)	DR grading	Public: FINDeRS 315 fundus images	CNN	Detection Accuracy: 95.71% Sensitivity: 76.92% Specificity: 100% Grading Accuracy: 60.28% Sensitivity: 65.40% Specificity: 73.37%	The network needs more data to train. And, this work opens up the future possibility to establish an integrated DR system to grade the severity at a low cost

ElTanboly et al. [66] (Mansoura University)	DR detection	Local: 52 SD-OCT scans	DFCN	AUC: 0.98 Accuracy: 92% Sensitivity: 83% Specificity: 100%	The proposed CAD system for early DR detection using the OCT retinal images has good outcome and outperforms than other 4 conventional classifiers

Prahs et al. [100] (Department of Ophthalmology, University of Regensburg)	Give an indication of the treatment of anti-VEGF injection	Local: 183,402 OCT B-scans	DCNN (GoogLeNet)	AUC: 0.968 Accuracy: 95.5% Sensitivity: 90.1% Specificity: 96.2%	The DCNN neural networks are effective in assessing OCT scans with regard to treatment indication with anti-VEGF medications

Abràmoff et al. [62] (University of Iowa Hospitals and Clinics)	DR detection	Public: Messidor 1748 fundus images	CNN	Referable DR: AUC: 0.980 Sensitivity: 96.8% Specificity: 87% Vision threatening DR: AUC: 0.989 Sensitivity: 100% Specificity: 90.8%	The DL enhanced algorithms have the potential to improve the efficiency of DR screening

Takahashi et al. [65] (Department of Ophthalmology, Jichi Medical University)	DR grading	Local: 9939 fundus images	DCNN (GoogLeNet)	Accuracy: 0.64∼0.82	The proposed novel AI disease-staging system have the ability to grade DR involving retinal areas not typically visualized on fundoscopy

Abbas et al. [64] (Surgery Department and Glaucoma Unity, University Hospital Puerta del Mar, Cádiz)	DR grading	Public: Messidor, DiaretDB, FAZ 500 fundus images Local: 250 fundus images	DNN	AUC: 0.924 Sensitivity: 92.18% Specificity: 94.50%	The system is appropriate for early detection of DR and provides an effective treatment for prediction type of diabetes

Chen et al. [73] (Institute for Infocomm Research, Agency for Science, Technology and Research; Singapore National Eye Centre)	Glaucoma detection	Public: Origa, Sces 2326 fundus images	DCNN	AUC: 0.831 for Origa 0.887 for Sces	Present a DL framework for glaucoma detection based on DCNN

Li et al. [89] (Institute for Infocomm Research, Agency for Science, Technology and Research)	Glaucoma detection	Public: Origa 650 fundus images	DCNN (AlexNet, VGG-19, VGG-16, GoogLeNet)	Best AUC: 0.8384 AlexNet > VGG-19 ≈ VG-16 > GoogLeNet	The proposed method that integrates both local and holistic features of optic disc to detect glaucoma is reliable

Asaoka et al. [91] (Department of Ophthalmology, The University of Tokyo)	Preperimetric OAG detection	Local: 279 VFs	DFNN	AUC: 92.6%	Using a deep FNN can distinguish preperimetric glaucoma VFs from healthy VFs with very high accuracy, which is better than the outcome obtained from ML techniques

Muhammad et al. [92] (Department of Physiology, Weill Cornell Medicine)	Glaucoma detection	Local: 612 single wide-field OCT images	DCNN (AlexNet)	Accuracy: 65.7%∼92.4%	The proposed protocol outperforms standard OCT and VF in distinguishing healthy suspect eyes from eyes with early glaucoma

Li et al. [90] (Zhongshan Ophthalmic Center, Sun Yat-sen University)	Glaucoma detection	Local: 8000 fundus images	DCNN (GoogleNet)	AUC: 0.986 Sensitivity: 95.6% Specificity: 92%	DL can be applied to detect referable glaucomatous optic neuropathy with high sensitivity and speciﬁcity

Burlina et al. [104] (Retina Division, Wilmer Eye Institute, Johns Hopkins University School of Medicine)	AMD Grading	Public: AREDS 5664 fundus images	DCNN	Accuracy 79.4% (4-class) 81.5% (3-class) 93.4% (2-class)	Demonstrates comparable performance between computer and physician grading

Burlina et al. [103] (Retina Division, Wilmer Eye Institute, Johns Hopkins University School of Medicine)	AMD detection	Public: AREDS 130000 fundus images	DCNN (AlexNet)	AUC: 0.94∼0.96 Accuracy: 88.4%∼91.6%	Applying a DL-based automated assessment of AMD from fundus images can produce results that are similar to human performance levels

Treder et al. [105] (Department of Ophthalmology, University of Münster Medical Center)	AMD detection	Local: 1112 SD-OCT images	DCNN	Sensitivity: 100% Specificity: 92% Accuracy: 96%	With the DL-based approach, it is possible to detect AMD in SD-OCT with good outcome. With more image data, the model can get more practical value in clinical decisions

Gao et al. [111] (Microsoft Research Asia and Singapore Eye Research Institute)	Nuclear cataracts grading	Public: ACHIKO-NC 5378 slit-lamp images	CNN and SVM	Accuracy: 70.7%	The proposed method is useful for assisting and improving diagnosis of the disease in the background of large-population screening and has the potential to be applied to other eye diseases

Long et al. [114] (Zhongshan Ophthalmic Centre, Sun Yat-sen University)	Pediatric cataracts detection	Local: CCPMOH 886 slit-lamp images	DCNN	Accuracy 98.87% (detection) 97.56% (treatment suggestion)	The AI agent using DL have the ability to accurately diagnose and provide treatment decisions for congenital cataracts. And the AI agent and individual ophthalmologists perform equally well. A cloud-based platform integrated with the AI agent for multihospital collaboration was built to improve disease management

Choi et al. [120] (Department of Ophthalmology, Yonsei University College of Medicine)	Multiple retinal diseases detection	Public: STARE 397 fundus images	DCNN (VGG-19)	Accuracy 30.5% (all categories were included) 36.7% (using ensemble classifiers) 72.8% (considering only normal, DR and AMD)	As the number of categories increased, the performance of the DL model has declined. Several ensemble classifiers enhanced the multicategorical classification performance. Large data sets should be applied to confirm the effectiveness of the proposed model
